# Repeated chemogenetic activation of C1 catecholamine neurons reduces subsequent glucoprivic responses and mimics HAAF

**DOI:** 10.1210/endocr/bqag074

**Published:** 2026-06-26

**Authors:** Ai-Jun Li, Qing Wang, Robert C Ritter, Suzanne M Appleyard

**Affiliations:** Programs in Neuroscience, Washington State University, Pullman, WA 99164-7620, USA; Programs in Neuroscience, Washington State University, Pullman, WA 99164-7620, USA; Programs in Neuroscience, Washington State University, Pullman, WA 99164-7620, USA; Programs in Neuroscience, Washington State University, Pullman, WA 99164-7620, USA

**Keywords:** catecholamines, HAAF, glucose regulation, epinephrine, pharmacogenetics, adrenal medulla

## Abstract

In diabetic patients, hypoglycemia-associated autonomic failure (HAAF) is a potentially lethal condition that results in attenuation of critical protective responses to low blood glucose, following repeated prior hypoglycemic episodes. The underlying mechanism(s) of HAAF is not fully understood. We previously demonstrated that activation of catecholamine (CA) neurons in the ventrolateral medulla (VLM) is both required and sufficient to elicit key protective responses to glucose deficit. Here we test the hypothesis that repeated selective activation of VLM CA neurons is sufficient to induce HAAF, even in the absence of glucose deficit. Using stereotaxic injections of an adeno-associated virus in female TH-Cre transgenic rats, we transfected rostral C1 CA neurons to express hM3D(Gq). In these rats, we found that a single clozapine-N-oxide (CNO) injection evoked robust hyperglycemia, with a magnitude and time course similar to those evoked by the glucoprivic agent, 2-deoxy-D-glucose (2DG). However, following repeated CNO injections in these same rats, the hyperglycemic response evoked by either CNO or 2DG were significantly attenuated. Moreover, plasma epinephrine levels, and Fos expression in VLM CA neurons and in the adrenal medulla, also were attenuated following repeated CNO injections. This constellation of effects is similar to those that define HAAF. Taken together our results suggest that repeated stimulation of VLM CA neurons mimics and could be a cause of the impairment of counterregulatory responses associated with pathogenesis of HAAF.

Glucose is the required metabolic fuel for cells in the brain, and glucose must be supplied to the brain from the blood because little glucose is stored in the brain itself. If glucose levels drop, or cellular glucose metabolism is impaired, a series of robust protective and corrective responses, known as counterregulatory responses (CRRs), are mobilized by glucosensing circuits in the brain to maintain its glucose supply. These CRRs include increased food intake, corticosterone secretion, and epinephrine release, resulting in an increase of glucose. Collectively, these responses and others are critical for maintaining the brain glucose supply (1-4). However, in diabetic patients, repeated episodes of cellular glucoprivation or insulin-induced hypoglycemia can impair subsequent CRRs, leading to a potentially lethal condition known as hypoglycemia-associated autonomic failure (HAAF) (5, 6). HAAF, which can result from inadvertent mismatches of blood glucose levels and insulin dose, is a major and potentially lethal threat to diabetic patients on insulin therapy. The pathophysiology of HAAF is poorly understood. However, studies in the 1970s and 1980s established that the key CRRs are triggered by activation of glucose-sensing neurons in the hindbrain (7-9). Moreover, recently published results have demonstrated that activation of C1 and A1 catecholamine (CA) neurons in the ventrolateral medulla (VLM) is necessary and sufficient for elicitation of key CRRs, including increased food intake, corticosterone secretion, and adrenal medullary epinephrine release to preserve and extend glucose supply (10-13). Therefore, we wanted to test the possibility that altered hindbrain CA neuron activity might participate in the pathogenesis of HAAF.

In the VLM, CA neurons distribute from caudal to rostral, as A1 (noradrenergic) and C1 (adrenergic) neurons with an overlap region (A1/C1). Early anatomical studies demonstrated that caudal C1 (A1/C1 overlap) CA neurons project to the hypothalamus, while rostral C1 CA neurons project to the spinal cord (14-16). Selective lesion of spinally projecting rostral C1 CA neurons abolishes the hyperglycemia evoked by adrenal medullary epinephrine release in response to glucoprivation, while selective lesion of rostrally projecting A1/C1 CA neurons that pass through or terminate in the paraventricular hypothalamic area abolishes glucoprivic feeding and corticosterone responses (10-12). Additionally, a recent report has further demonstrated the contribution of a CA/neuropeptide Y projection from the VLM to the perifornical lateral hypothalamic area in glucoprivic feeding (17). Although these data support a role for VLM CA neurons in the elicitation of CRRs, it remains unclear whether repeated activation of these neurons contributes to HAAF. Systemically administered insulin or antiglycolytic drugs, such as 2-deoxy-D-glucose (2DG), are most commonly used to study glucose counterregulation. Systemic insulin and 2DG produce glucoprivation and also cause large metabolic changes throughout the body and brain. Hence, it is difficult to discern whether HAAF, induced by systemic 2DG or insulin injection, results from desensitization or reduced responsiveness of a specific neuronal population, such as VLM CA neurons, or is the result of a more dispersed or distal effect of glucoprivation. To overcome some of these problems, we took advantage of chemogenetic tools and transgenic rats, allowing us to selectively activate CA neurons in VLM subregions.

In this study we injected a Cre-dependent DREADD (designer receptor exclusively activated by designer drugs) construct unilaterally into the rostral C1 regions of TH-Cre^+^ transgenic rats to express hM3D(Gq) receptor selectively in CA neurons in this region. We then were able to selectively activate the transfected CA neurons repeatedly using systemic injections of the DREADD agonist, clozapine-N-oxide (CNO), in the absence of glucoprivation. Our results indicate that prior selective and repeated chemogenetic activation of rostral C1 CA neurons, even in the absence of glucoprivation, significantly reduces the subsequent CRRs and CA neuron and adrenal medullary activation by either CNO or 2DG. In other words, we found that repeated CNO activation of C1 CA neurons mimicked components of HAAF, including attenuated hyperglycemic response, reduced epinephrine release, as well as reduced Fos expression in VLM CA neurons and in adrenal medullary cells, following subsequent acute glucoprivation. These findings support the hypothesis that repeated activation of CA neurons is sufficient for the impairment of CRRs that are pathognomonic of HAAF.

## Materials and methods

### Animals and genotyping

Female heterozygous transgenic rats (Long-Evans gene background) expressing Cre recombinase under the control of tyrosine hydroxylase (TH) promoter (Long-Evans-Tg (TH-Cre) 3.1) and their wild-type non-Cre littermates (TH-Cre^+^ and TH-Cre^−^ rats, respectively) were used in the present study (18). These transgenic rats were bred in our vivarium from breeding stock generously provided by Dr Karl Deisseroth (Stanford University) and were age 4 months at the beginning of the experiments. Genotyping was performed from an ear punch at age 3 weeks using polymerase chain reaction (19). Rats were maintained on a 12-hour light/12-hour dark cycle (lights on at 7 Am) with ad libitum access to pelleted rodent food (catalog No. 5001, LabDiet) and tap water. All experimental procedures conformed to National Institutes of Health (NIH) guidelines and were approved by the Washington State University Institutional Animal Care and Use Committee.

### Viral injection

Rats were anesthetized using 1.0 mL/kg of a ketamine/xylazine/acepromazine cocktail (50 mg/kg ketamine HCl, Fort Dodge Animal Health; 5.0 mg/kg xylazine, Vedco; 1.0 mg/kg acepromazine, Vedco) and placed in a stereotaxic device for intracranial injection of adeno-associated virus (AAV). They were injected with an AAV (serotype 2) containing a Cre-dependent doubly floxed inverted open reading frame encoding a DREADD receptor and a reporter gene (mCherry) under a human synapsin I promotor, AAV2-DIO-hSyn-hM3D(Gq)-mCherry (hM3D(Gq); 1.6 × 10^12^ particles/mL, catalog No. 44361, RRID Addgene_44361, Addgene). Following transfection with this vector, the DREADD receptor is selectively expressed only by Cre-expressing CA neurons near the injection site. To achieve a broad expression hM3D(Gq) in caudal C1, AAV injection (200 nL/site) was delivered unilaterally into two spots: one at the medial portion of C1 (C1m) and another at a more rostral portion of C1 (C1r) (both on the right side; coordinates described later) through a pulled glass capillary pipette (30-µm tip diameter) using a Picospritzer (Parker). Pipettes entered the brain just dorsolateral to the targeted sites and were driven ventromedially at a 14° angle to avoid inadvertent damage to or transfection of CA neurons in the dorsomedial medulla by a potential diffusion of the AAV along the pipette tract (19). The coordinates for C1m (and C1r) were 12.95 (12.25) mm caudal to the bregma; 4.0 (4.0) mm lateral to the midline, and 8.4 (8.5) mm ventral to the skull surface (20).

Previously published (19, 21) and unpublished observations from our laboratory indicate that mCherry expression in CA neurons is maximal and stable between 5 and 20 weeks after this virus injection. TH-Cre^−^ rats with hM3D(Gq) injection into VLM showed no mCherry expression in the injection site, verifying that expression of the construct was Cre dependent. In addition, to ensure that CNO effects were dependent on activation of transfected CA neurons, CNO was tested in TH-Cre^−^ rats injected in the VLM with the same DREADD construct and in nontransfected TH-Cre^+^ rats. Neither of these control treatments increased blood glucose levels.

### Experiment 1. Effect of repeated clozapine-N-oxide or 2-deoxy-D-glucose injection on blood glucose

All tests for this study were conducted beginning at 5 and up to 17 weeks after AAV injection into VLM. The present study and our unpublished data demonstrate stable mCherry expression in VLM CA neurons at 12, 17, and up to 20 weeks after AAV injection. We also saw similar CNO-induced food intake and hyperglycemia in rats perfused more than 12 weeks compared with less than 12 weeks after AAV injections. At 5 weeks after AAV injection, rats were screened in a CNO-induced hyperglycemia test to determine the efficacy of the DREADD transfection. Rats with a hyperglycemia after CNO injection greater than 30% increase from the baseline during the 4-hour test were randomly divided into different groups for further testing, as described later. Tests were performed every 3 weeks to avoiding potential toleration to injected drugs. Each rat was injected once daily for 4 days with CNO (1 mg/kg, intraperitoneally [i.p.]; catalog No. HB6149, Hello Bio) or 2DG (200 mg/kg, subcutaneously [s.c.]; catalog No. 14325, Cayman Chemical), doses shown to produce hyperglycemic responses of similar magnitudes to those observed in our preliminary experiments, or with saline (Sal) as control (0.9%, i.p. or s.c.). On days 1 to 4, food was removed 2 hours before and returned 4 hours after injection. On day 5, each rat was tested with either the same or a different drug so that the resulting groups were as follows: rSal-Sal, rSal-CNO, rCNO-CNO, r2DG-CNO, rSal-2DG, r2DG-2DG and rCNO-2DG (r for repeated). For tests on day 5, food was removed 2 hours prior to the injection and then blood glucose levels were measured just before and 1, 2, and 4 hours after the injection, with a glucose monitor (Contour Next, Bayer) from tail blood drops.

At the end of the experiment, specificity and efficacy of the transfected DREADD construct in VLM were confirmed by immunohistochemical (IHC) analysis of mCherry and CA biosynthetic enzyme, dopamine-β hydroxylase (DBH), as described in Experiment 2.

### Experiment 2. Effect of repeated clozapine-N-oxide injection on Fos expression in the hindbrain and adrenal medulla

In this experiment, rats were screened at 5 weeks after C1m and C1r unilateral hM3D(Gq) injection for Sal- and CNO-induced hyperglycemia, as described in Experiment 1. Rats with good hyperglycemia responses (>30% over baseline at 1 hour or 2 hours after CNO injection) were then randomly divided into different groups and injected once daily for 5 days with Sal, CNO, or 2DG, as described in Experiment 1. On day 5, rats were euthanized and perfused 90 minutes after the injection and brain and adrenal medulla were collected for immunostaining of Fos, mCherry and DBH.

#### Perfusion

For IHC staining, rats were euthanized by deep isoflurane-induced anesthesia (Halocarbon Products). Just prior to cessation of the heartbeat, rats were perfused transcardially with phosphate-buffered saline (PBS) (pH 7.4), followed by freshly made 4% formaldehyde/PBS solution. Brains were rapidly removed and placed in 4% formaldehyde/PBS for another 5 hours, followed by immersion overnight in 25% sucrose in PBS at room temperature. Brains were sectioned in the coronal plane at 40-µm thickness (4 serial sets) and adrenal medulla at 40-µm thickness (2 serial sets) using a cryostat.

#### Immunohistochemistry of brain sections

For double immunofluorescence staining, brain sections were incubated with primary antibodies at 4°C (2.5 days) in 10% normal horse serum (NHS)/PBS, washed, and then incubated in secondary antibodies (4 hours) at room temperature (19). The following primary antibodies were used: guinea pig anti-RFP (1:12 000 dilutions; to detect mCherry; catalog No. 390004, RRID AB_2737052, Synaptic systems), mouse anti-DBH (1:5000 dilutions; to detect CA neurons; catalog No. MAB308, RRID AB_2245740, Millipore-Sigma), and rabbit anti-Fos (1:1000 dilution; to detect activated neurons; catalog No. ZRB457, RRID AB_2934037, Millipore-Sigma). Secondary antibodies were donkey anti-mouse, anti-rabbit, or anti-guinea pig, conjugated with Alexa 488 or Cy3 (all diluted 1:500 in 1% NHS/PBS; Jackson ImmunoResearch). Sections were mounted and cover-slipped with ProLong-Gold medium (catalog No. P36934, ThermoFisher Scientific), then examined and photographed using a Zeiss epifluorescent microscope.

#### Hindbrain subregions and Fos cell counting in the ventrolateral medulla

Catecholamine cell groups in the hindbrain are defined as described in *The Rat Brain in Stereotaxic Coordinates* (20). Cells of groups C1 and A1 are continuously distributed along the rostrocaudal extent of the VLM. We refer to the overlap of caudal C1 with rostral A1 as A1/C1 (14.1-13.4 mm caudal to bregma), the medial portion of C1 as C1m (13.3-12.5 mm caudal to bregma), and the rostral portion of C1 as C1r (12.4-11.8 mm caudal to bregma). To count the numbers of cells with positive immunostaining of DBH, Fos and mCherry, 2 sections for C1m or C1r were counted from each rat and were averaged.

#### Immunohistochemical staining and quantification of Fos in the adrenal medulla

To visualize the expression of Fos in the adrenal medulla, IHC was performed using standard avidin-biotin-peroxidase techniques as previously described (22). Adrenal medulla sections were first incubated in rabbit anti-Fos (1:1000 dilution; catalog No. ZRB457, RRID AB_2934037, Millipore-Sigma) diluted in 10% NHS in PBS for 2 days. After an overnight incubation in biotin-conjugated donkey anti-rabbit immunoglobulin G (1:500 dilution; catalog No. 711-065-152, RRID AB_2340593, Jackson ImmunoResearch), washing, then incubation for 4 hours in ExtrAvidin-Peroxidase (1:1500 dilution in PBS; catalog No. E2886, RRID AB_2620165, Millipore-Sigma), and another washing; diaminobenzidine was used in the peroxidase reaction to produce a brown reaction product for Fos labeling. Pictures of Fos staining in the adrenal medulla were taken from 2 areas of adrenal medulla per section, in each of 3 sections per rat. The intensity of Fos staining above background was measured in these 6 images using ImageJ software (NIH) and was averaged for each rat.

### Experiment 3. Effect of repeated clozapine-N-oxide injection on epinephrine levels

In this experiment, rats were screened for Sal and CNO injection on blood glucose 5 weeks after DREADD construct injection into C1m and C1r, as in Experiment 1, then implanted with a jugular catheter and adapted to the blood collection protocol. Rats were injected once daily with Sal, CNO, or 2DG for 5 consecutive days in the absence of food as in Experiments 1 and 2. On the fifth day, blood samples were collected by remote withdrawal from the implanted catheters.

#### Jugular catheter implantation, blood sample collecting, and epinephrine enzyme-linked immunosorbent assay

After screening for CNO-induced hyperglycemia, rats were implanted with chronic jugular catheters for remote blood collection to minimize handling stress-induced changes on epinephrine measurement during sample collection. The catheter, made of Silastic tubing (inside diameter, 0.64 mm; outside diameter, 1.19 mm; Dow Corning), was guided into the right cardiac atrium through the right jugular vein under ketamine/xylazine/acepromazine anesthesia, as previously described (12). After recovery from surgery, the rats were habituated (4 hours daily for 1 week) to 30-cm × 10-cm opaque Plexiglas chambers, which were used for the remote blood sampling. Then rats were separated into different groups and received daily injection for 5 days as in the aforementioned experiments. On the fifth day, blood samples (∼600 µL each) were collected remotely via the implanted catheters at 30 minutes before and 1 hour after each injection. Blood samples were allowed to clot for 1 to 2 hours at 4 °C. The blood then was centrifuged, and serum samples were transferred to fresh tubes and stored at −80 °C. Serum epinephrine concentrations were measured using an enzyme-linked immunosorbent assay kit (catalog No. IB89539R, IBL America) according to the provided instructions. Blood glucose levels were also measured during blood sample collection using a Contour Next glucose monitor.

At the conclusion of the experiment, rats were prepared for IHC analysis of mCherry and DBH, as described earlier in Experiment 2, to verify placement, specificity, and efficacy of the transfected DREADD construct.

### Statistics

All results are presented as mean ± SEM. One-way or 2-way repeated-measures analyses of variance were used for statistical analysis of data. After statistical significance was determined, multiple comparisons between individual groups were tested by a post hoc Student-Newman-Keuls test. A *P* less than .05 was considered to be statistically significant.

## Results

### Repeated clozapine-N-oxide or 2-deoxy-D-glucose injections reduced subsequent CNO- or 2DG-induced hyperglycemia

**Figure 1:**
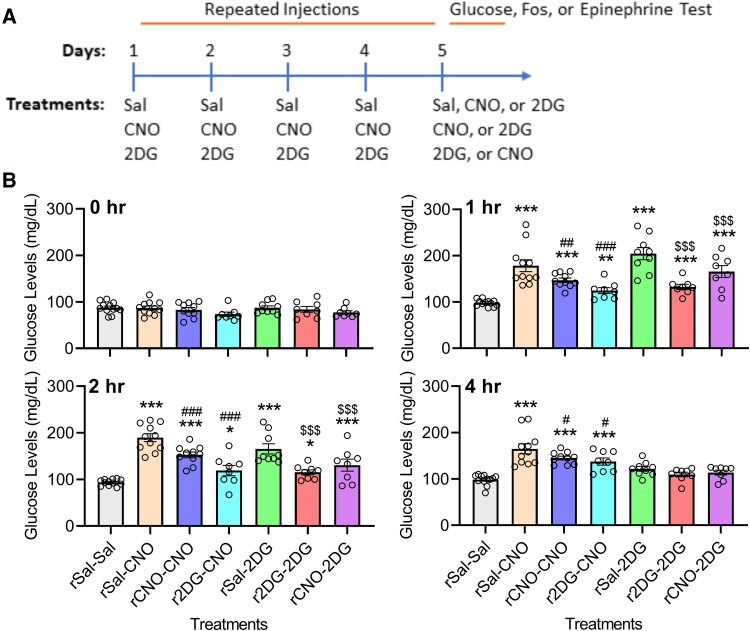
Blood glucose levels after repeated clozapine-N-oxide (CNO) and 2-deoxy-D-glucose (2DG) injections. A, Timeline for all treatments in this study. Rats were received repeated daily injection of saline (Sal), CNO, or 2DG for 4 days. On test day (day 5), rats were injected with Sal, CNO or 2DG, and blood glucose (Experiment 1), Fos expression in the brain and adrenal medulla (Experiment 2) or blood epinephrine (Experiment 3) was measured. B, Data of repeated CNO or 2DG on blood glucose from Experiment 1. Female C1m-C1r^hM3D(Gq)^ rats, transfected unilaterally with AAV2-DIO-hSyn-hM3D(Gq)-mCherry (hM3D(Gq)) into C1m and C1r, were injected daily with Sal (i.p. or s.c.), CNO (1 mg/kg; i.p.), or 2DG (200 mg/kg; s.c.) for 5 days in the absence of food (r for repeated injection of first 4 days with same chemical). On day 5, blood glucose levels were measured just before (0 hour) and 1 hour, 2 hours, and 4 hours after the injection. *, **, ***P less than .05, .01, .001 (vs rSal-Sal); #, ##, ###*P* less than .05, .01, .001 (vs rSal-CNO); $$$*P* less than .001 (vs rSal-2DG) (Student-Newman-Keuls test after 2-way repeated-measures analysis of variance). N = 8-12 rats/group.

A timeline indicating the order of treatments in all 3 experiments reported here is shown in Fig. 1A. In Experiment 1, the effects of repeated CNO or 2DG injections on CNO- or 2DG-induced hyperglycemia response were investigated. As shown in Fig. 1B, single CNO injection following repeated daily Sal injections (rSal-CNO) in C1m-C1r^hM3D(Gq)^ rats significantly increased blood glucose at 1 hour, 2 hours, and 4 hours after CNO injection when compared to Sal-injected (rSal-Sal) rats (*P*s < .001). Single injection of 2DG following repeated Sal injections (rSal-2DG) also produced a hyperglycemia response that was similar in magnitude and time course to that produced by single CNO injection. In contrast, daily injections of CNO for 4 days reduced the CNO-induced hyperglycemia on day 5 at all time points measured (*P*s < .05; rCNO-CNO vs rSal-CNO group). Similarly, 4 daily 2DG injections reduced the 2DG-induced hyperglycemia on day 5 (*P*s < .001; r2DG-2DG vs rSal-2DG group). This loss in glucoprivic response was the same between CNO and 2DG as repeated 2DG injections on days 1 to 4 reduced the CNO-induced hyperglycemia on day 5 (*P*s < .05; r2DG-CNO vs rSal-CNO group). Conversely, repeated 4-day CNO injections reduced subsequent 2DG-induced hyperglycemia (*P*s < .01; rCNO-2DG vs rSal-2DG group). Body weights did not change significantly during these 5 days between the groups (not shown).

### Repeated clozapine-N-oxide or 2-deoxy-D-glucose injections inhibited CNO- or 2DG-induced Fos expression in the hindbrain

In Experiment 2 we examined the effect of repeated activation of C1m-C1r CA neurons by CNO or 2DG on Fos expression in the hindbrain and in the adrenal medulla.

**Figure 2:**
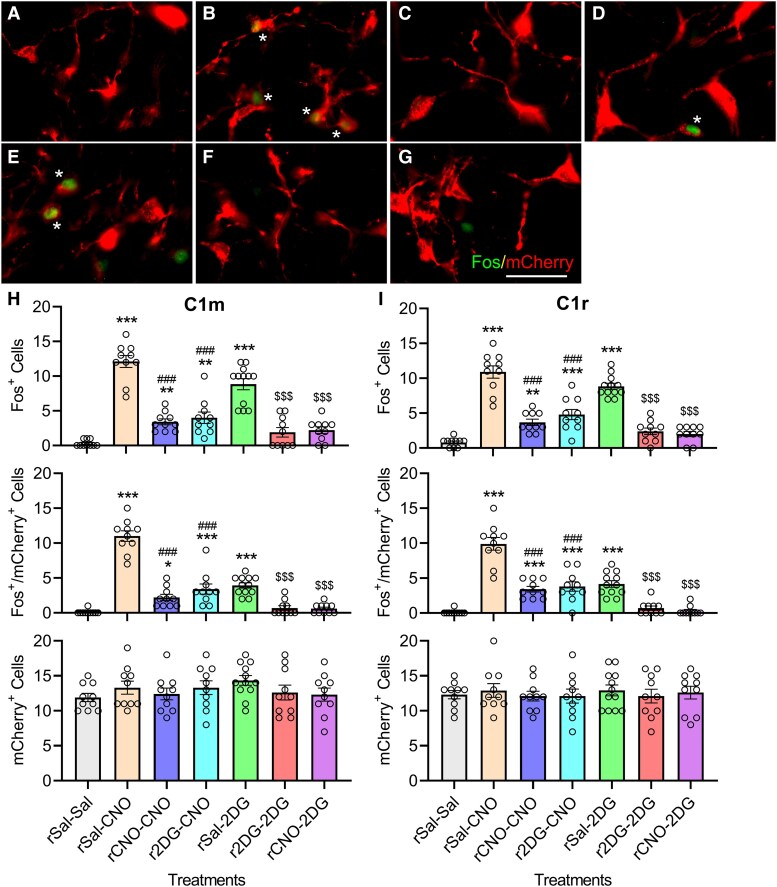
Effects of repeated clozapine-N-oxide (CNO) and 2-deoxy-D-glucose (2DG) injections on Fos expression in the hindbrain. Data from cohort of Experiment 2. Female C1m-C1r^hM3D(Gq)^ rats were injected daily with saline (Sal), CNO, or 2DG for 5 days as in Fig.1. On day 5, brain tissues were collected 90 minutes after the injection for immunohistochemistry. A to G, Representative images of Fos (green) and mCherry (red) expressions in hM3D(Gq)-transfected C1m area from rSal-Sal, rSal-CNO, rCNO-CNO, r2DG-CNO, rSal-2DG, r2DG-2DG, or rCNO-2DG treated rats, respectively. Asterisks indicate double-stained cells. Bar, 50 µm. H and I, Counts of Fos-, Fos/mCherry-, and mCherry-positive cells in the hM3D(Gq)-transfected C1m (H) and -C1r (I) region. *, **, ***P less than .05, .01, .001 (vs rSal-Sal); ###*P* less than .001 (vs rSal-CNO); $$$*P* less than .001 (vs rSal-2DG) (Student-Newman-Keuls test after 1-way analysis of variance). N = 5-6 rats/group (counted 2 sections for each rat each region).

**Figure 3:**
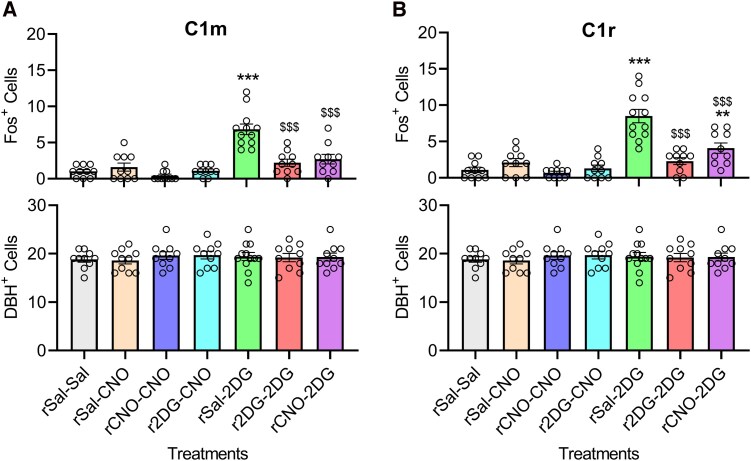
Effects of repeated clozapine-N-oxide (CNO) and 2-deoxy-D-glucose (2DG) injections on Fos expression in non–adeno-associated virus (non-AAV) injection side of the hindbrain. Data from cohort of Experiment 2 as in Fig. 2. Cell counts of Fos-, and DBH-positive cells in the non–AAV-injected -C1m (A) and C1r (B) sides from rSal-Sal–, rSal-CNO–, rCNO-CNO–, r2DG-CNO–, rSal-2DG–, r2DG-2DG–, or rCNO-2DG–treated group, respectively. **, ***P less than .01, .001 (vs rSal-Sal); $$$*P* less than .001 (vs rSal-2DG) (Student-Newman-Keuls test after 1-way analysis of variance). N = 5-6 rats/group (counted 2 sections for each rat each region).

In the hindbrain, after a repeated Sal injection, a single injection of CNO increased Fos- and Fos/mCherry-positive cell numbers in the C1m and C1r region of the AAV-injected side (Fig. 2). After repeated CNO injections for 4 consecutive days, the total Fos- and Fos/mCherry-positive cell numbers in C1m and C1r of hM3D(Gq)-transfected sides were dramatically decreased in rCNO-CNO rats compared to rSal-CNO rats on day 5 (*P*s < .001). Repeated 2DG injections also significantly decreased 2DG-induced Fos and Fos/mCherry expression in C1m and C1r on the AAV-injected side (*P*s < .001). Similar inhibitions were observed in r2DG-CNO (on the AAV-injected side) and rCNO-2DG (on the AAV-injected side) groups when compared to rSal-CNO and rSal-2DG, respectively (*P*s < .01). There was no difference in cell numbers of mCherry-positive cells between groups in the AAV-injected C1m and C1r regions. In contrast, on the non–AAV-injected contralateral sides of C1m and C1r, single or repeated CNO injection had no effect on Fos expression in CA neurons (Fig. 3). A single 2DG injection increased Fos expression in C1m and C1r neurons on the non–AAV-injected contralateral sides. Repeated 2DG injections led to reduced induction of Fos expression on the non-AAV contralateral sites both in the C1m and C1r regions (*P*s < .001). Importantly, repeated CNO injection also decreased the 2DG-induced Fos expression on the non-AAV C1m and C1r sides (*P*s < .001; rCNO-2DG vs rSal-2DG group).

### Repeated clozapine-N-oxide or 2-deoxy-D-glucose injections inhibited CNO- or 2DG-induced Fos expression in the adrenal medulla

**Figure 4:**
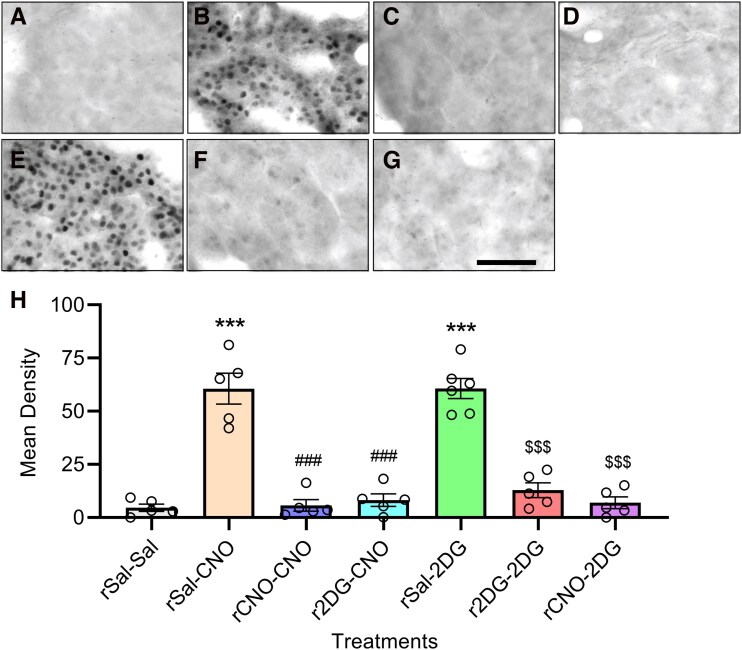
Effects of repeated clozapine-N-oxide (CNO) and 2-deoxy-D-glucose (2DG) injections on Fos expression in the adrenal medulla. Data from cohort of Experiment 2 as in Fig. 2. A to G, Representative images of Fos expression in adrenal medulla from a rSal-Sal–, rSal-CNO–, rCNO-CNO–, r2DG-CNO–, rSal-2DG–, r2DG-2DG–, or rCNO-2DG–treated rat, respectively. Bar, 50 µm. H, Quantification of Fos expression for each group. ***P less than .001 (vs rSal-Sal); ###*P* less than .001 (vs rSal-CNO); $$$*P* less than .001 (vs rSal-2DG) (Student-Newman-Keuls test after 1-way analysis of variance). N = 5-6 rats/group (mean density for each rat was measured and averaged in 2 regions/section from 3 sections per rat).

Fos expression in the adrenal medulla was also examined in Experiment 2 (Fig. 4). Single injection of CNO (rSal-CNO) or 2DG (rSal-2DG) significantly enhanced Fos expression in the adrenal medulla under control conditions (*P*s < .001). In contrast, repeated CNO or 2DG injections for 4 days blunted the ability of either 2DG or CNO to induce Fos expression in the adrenal medulla on day 5 (*P*s < .001).

### Repeated clozapine-N-oxide or 2-deoxy-D-glucose injections inhibited CNO- or 2DG-induced epinephrine release

**Figure 5:**
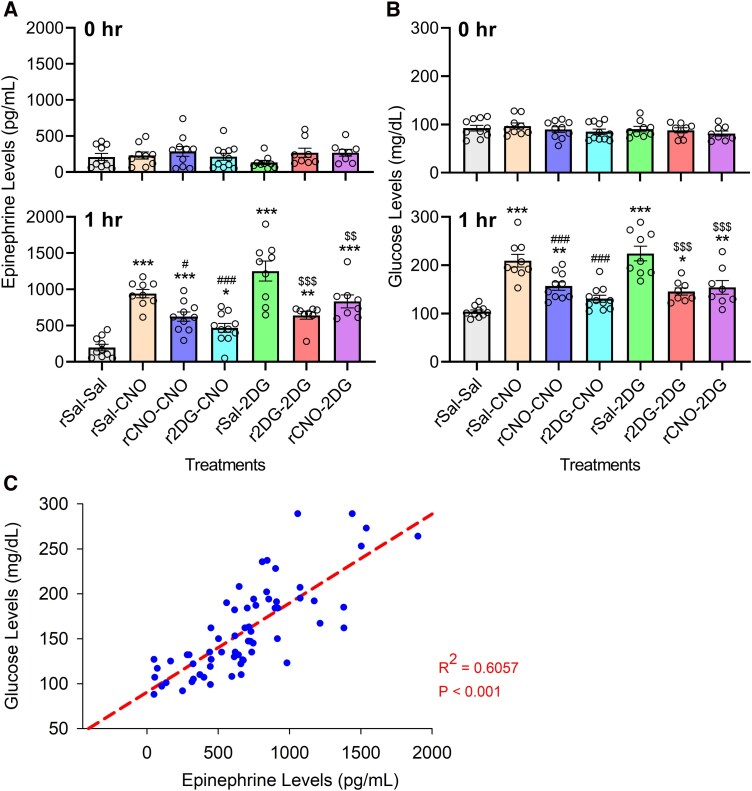
Effects of repeated clozapine-N-oxide (CNO) and 2-deoxy-D-glucose (2DG) injections on blood epinephrine and glucose levels. In Experiment 3, female C1m-C1r^hM3D(Gq)^ rats were injected daily with saline (Sal), CNO, or 2DG as in Fig. 1. On day 5, blood glucose and epinephrine levels were measured just before (0 hour) and 1 hour after the injection. A, Changes of blood epinephrine levels on day 5. B, Changes of blood glucose levels on day 5. *, **, ***P less than .05, .01, and .001 (vs rSal-Sal); #, ###*P* less than .05, .001 (vs rSal-CNO); $$, $$$*P* less than .01, .001 (vs rSal-2DG) (Student-Newman-Keuls test after 2-way repeated-measures analysis of variance). N = 8-11 rats/group. C, Scatter plot of glucose and epinephrine. Correlation of blood glucose and epinephrine levels from all C1m-C1r^hM3D(Gq)^ rats in this experiment showed strong positive correlation. Correlation *R* value and statistical significance are presented.

In Experiment 3 we wanted to examine whether repeated activation of C1m-C1r CA neurons by CNO or 2DG also altered the CRRs to increase epinephrine release in the blood. As shown in Fig. 5A and 5B, single injection of CNO (rSal-CNO) or 2DG (rSal-2DG) significantly increased epinephrine and glucose levels in the blood (*P*s < .001). Four days of CNO or 2DG injections in C1m-C1r^hM3D(Gq)^ rats significantly inhibited the ability of a CNO or 2DG injection on day 5 to induce epinephrine release and blood glucose levels (*P*s < .05). A scatter plot of glucose and epinephrine levels for all rats is shown in Fig. 5C. Correlational analysis of glucose and epinephrine levels showed a strong positive correction (*R*^2^ = 0.6057 and *P* < .001).

### Viral transfection in the hindbrain

**Figure 6:**
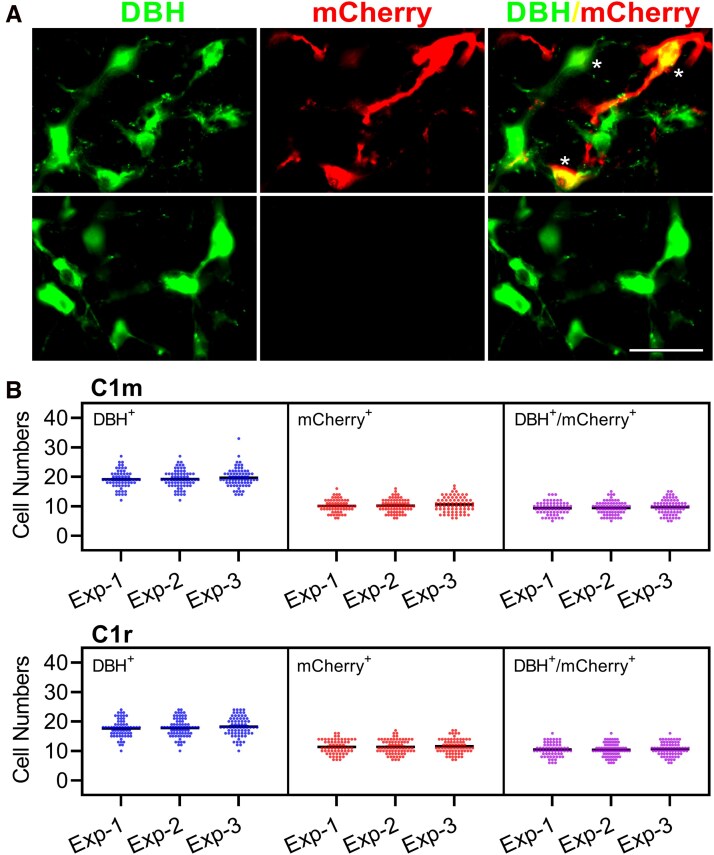
Dopamine-β hydroxylase (DBH) and mCherry expressions in the hindbrain. Transfection of hM3D(Gq) were determined by counting DBH/mCherry cells in the adeno-associated virus (AAV)-injected sites at the end of each experiment. A, Representative images of DBH (green) and mCherry (red) expression in the C1m area from an hM3D(Gq)-transfected rat. Upper panels, AAV-injected side; lower panels, non–AAV-injection side. Asterisks indicate double-stained cells. Bar, 50 µm. B, Averages of cell count of DBH-, mCherry- and DBH/mCherry-positive cells in the hM3D(Gq)-transfected C1m and C1r sides from rats used in each experiment.

At the end of all the experiments, hM3D(Gq) transfection in CA neurons was confirmed by double staining of DBH and mCherry in the hindbrain for all rats used in the experiments. Typical examples of DBH and mCherry expression is shown in Fig. 6A. Transfected cells were distributed in C1m and C1r in proximity to the injection site, where nearly half (49%-59%) of the CA neurons in C1m and C1r were transfected for each experiment (Fig. 6B), and there was no difference between the experiments. These numbers were also similar to that reported in our previous studies (19, 21, 22). A total of 91% to 93% of the mCherry-positive cells were also DBH positive in transfected C1m and C1r regions, and there was also no difference between the experiments (see Fig. 6B). A detailed analysis of these two numbers in Experiment 2 revealed no difference between treatment groups (not shown). In addition, 92% and 90% of Fos-positive cells in C1m and C1r after CNO injection in rSal-CNO rats were also mCherry positive in Experiment 2. Together, these results demonstrated a specific transfection of hM3D(Gq) in Cre-expressing CA neurons and CNO injection selectively activated those cells. A pretest of CNO-induced hyperglycemia for all rats in the experiments showed that after CNO injection, blood glucose levels were increased from 94.1 ± 1.1 to 135.1 ± 3.4 at 1 hour, 170.8 ± 5.7 at 2 hours, and 157.0 ± 4.3 mg/dL at 4 hours after the injection.

## Discussion

Our results indicate that repeated, selective, chemogenetic activation of rostral C1 (C1m and C1r) CA neurons by CNO for 4 days in DREADD-transfected TH-Cre^+^ female rats reduced the counterregulatory adrenomedullary epinephrine release and the ensuing hyperglycemic response to acute glucoprivation. We also found that repeated CNO activation of C1 CA neurons attenuated glucoprivation-induced Fos expression in these VLM CA neurons and in the adrenal medullary chromaffin cells in response to subsequent injections of either CNO or 2DG. The effects of repeated CNO treatment on these key CRRs in the absence of prior glucoprivation were similar to those produced by repeated 2DG, thereby mimicking components of HAAF. These results are consistent with our previous work demonstrating the reduction of subsequent feeding, corticosterone secretion, and Fos expression in VLM after repeated chemogenetic activation of caudal C1 (A1/C1 overlap) CA neurons by CNO in DREADD-transfected TH-Cre^+^ male rats (21). Together, the fact that CA neuron activation, in the absence of glucoprivation, resulted in CRR attenuation in both studies and sexes strongly implicates the prior intense activation of CA neurons in the pathogenesis of HAAF.

In the present study, nearly half of CA neurons near the AAV injection sites were transfected with hM3D(Gq) as inferred from coexpression of the AAV reporter gene mCherry and the CA synthesizing enzyme, DBH. This percentage is consistent with our previous reports when the same AAV, at similar titers and volumes, was injected into C1 subregions in male and female TH-Cre^+^ rats (19, 22). The hM3D(Gq) expression was specific to CA neurons, since almost all of the mCherry-positive cells were also DBH positive in the AAV-infused CA regions and also virtually all of the Fos-positive cells in C1m and C1r after CNO treatment were mCherry-positive in the rSal-CNO group. Therefore, we conclude that CNO injection is sufficient to selectively activate CA neurons and initiate the related neuronal and behavioral events.

Several lines of evidence support the importance of CA neurons in the mediation of critical CRRs. Glucoprivation activates hindbrain CA neurons detected as increased Fos expression predominantly in the VLM (23). Indeed, disinhibition of spinally projecting rostral VLM (RVLM) neurons, which includes a group of 2DG-sensitive, nonbarosensitive neurons, induce hyperglycemia, and adrenalectomy significantly reduced this hyperglycemic response in rats (24). Selective immunotoxin lesion of specific VLM CA neuron groups abolishes CRRs, including increased food intake, blood glucose, plasma epinephrine and corticosterone release (11, 12). Specifically, some VLM CA neurons project rostrally to the hypothalamus and others project caudally to the spinal cord (14-16). Previous studies revealed that the spinal cord–projecting CA neurons in rostral C1 mediate the adrenal medullary response to glucoprivation. Immunotoxin-induced lesion of spinal cord–projecting C1r CA neurons abolish 2DG-induced hyperglycemia but not 2DG-induced hyperphagia (10, 11). Selective lesioning of spinally projecting CA neurons also abolishes increased Fos expression in the adrenal medulla following glucoprivation (11). In the present study, repeated chemogenetic activation of rostral (spinally projecting) C1 CA neurons, even in the absence of glucoprivation, attenuated adrenal medullary epinephrine release and consequent increase in blood glucose following either CNO administration or 2DG-induced glucoprivation. Importantly, increased Fos expression in hindbrain CA neurons, as well as in the adrenal medulla, also were attenuated after repeated VLM CA neuron activation, which is consistent with the interpretation that reduced activation of CA neurons is responsible for attenuation of CRRs. It is important to note that repeated CNO injections reduced the glucoprivic adrenal medullary responses both to subsequent CNO or 2DG injection. Similarly, repeated 2DG injections inhibited both 2DG- and CNO-induced glucoprivic adrenal medullary responses. This congruency of effects supports our hypothesis that the same mechanism, located in VLM CA neurons, is responsible for attenuation of CRRs following both systemic glucoprivation and localized CNO injection. Moreover, these results lead to the strong inference that decreased VLM CA neuron activity plays a pivotal role in the development of HAAF.

In contrast to the rostral C1 CA neurons, caudal VLM CA neurons project to the hypothalamus (14-16). This C1-hypothalamus pathway is required for increased feeding and corticosterone secretion in response to glucoprivation (10, 11). Our previous work, using the same DREADD approach that we used in the present study, demonstrated that repeated chemogenetic activation of caudal C1 (A1/C1) CA neurons in male rats results in attenuations of glucoprivic feeding and corticosterone secretory responses (21). It is well known that prior recurrent hypoglycemia or hyperinsulinemia attenuates CRRs in response to a subsequent hypoglycemia episode and causes HAAF, which comprises a wide range of pathophysiological conditions, including reduced epinephrine response, reduced sympathetic neural responses, and hypoglycemia unawareness (5, 6). Together with the observations reported in this paper, our previous results emphasize the critical role both of caudal and rostral VLM CA neurons in the pathogenesis of HAAF.

Although we believe that our evidence for a primary role for decreased CA neuron activity in the pathogenesis of HAAF is strong, we cannot rule out contributory roles of other nodes in the CRR circuitry in the pathogenesis of HAAF. For example, future studies involving direct and repeated activation on intermediolateral column neurons of the spinal cord and/or adrenal medulla cells might provide important additional clues to the pathogenesis of HAAF, as well as possible pharmaceutical targets for the treatment and prevention of HAAF disorder.

Counterregulatory hormones released during glucose deficits have also been implicated in the development of HAAF. Epinephrine is a key counterregulatory hormone necessary to restore glucose levels through eliciting robust hepatic glycogenolysis, mobilizing gluconeogenic substrates, and stimulating lipolysis (25). A loss of these effects has been suggested as a possible mechanism to cause HAAF (26, 27). Experimentally, CRRs to glucose deficit are mainly produced by insulin-induced hypoglycemia or by systemic or central administration of antiglycolytic drugs (10, 12, 23, 28), both of which cause a rise of blood epinephrine (29-32). Indeed, correlational analysis of glucose and epinephrine levels in the present study showed a strong positive correction when we pooled the data from repeated and single CNO- and 2DG-injected rats together. Recurrent hypoglycemia by insulin clamp leads to HAAF in humans, with blunted counterregulatory epinephrine release to subsequent hypoglycemia episodes (26, 29). Infusion of nonselective adrenergic antagonists prevents HAAF in nondiabetic humans (26). Furthermore, repeated infusion of epinephrine itself reduces epinephrine response to subsequent hypoglycemia in nondiabetic humans (27). These studies suggest that adrenergic activation may underlie the effect of a recent hypoglycemia to reduce the adrenal medullary response to subsequent hypoglycemia, the key feature of HAAF in diabetes. However, whether epinephrine and adrenergic receptors mediate the effect seen in the present study needs to be studied further.

Some investigators have suggested cortisol/corticosterone as a possible systemic mediator in the pathogenesis of HAAF. Cortisol infusion, like bouts of hypoglycemia, reduces adrenomedullary epinephrine and muscle sympathetic nerve activity responses to hypoglycemia in heathy humans (33). Cortisol injected into the third ventricle (34) or dexamethasone, a specific type II glucocorticoid agonist injected into the fourth ventricle (35), induced a similar reduction in the sympathoadrenal responses to subsequent hypoglycemia in rats. However, glucocorticoid actions as a mediator in the pathogenesis of HAAF remain controversial, since other reports have not observed reduced sympathoadrenal or hypoglycemic responses following cortisol or corticosterone administered either systemically or centrally in human or rodents (36-38). We have previously shown that blood corticosterone levels are increased by selectively activating caudal (A1/C1 or C1m), but not rostral (C1r) VLM CA neurons (19, 21). Furthermore, repeated activation of bilateral A1/C1 or C1m CA neurons daily for 5 days attenuated CNO- or 2DG-induced corticosterone release. However, the adrenomedullary glucose mobilization was not diminished following repeated CNO injections in rats with hM3D(Gq) transfected at these caudal C1 sites (21), in contrast to our present findings looking at the rostral sites. This supports the hypothesis that the more rostral C1 neurons underlie the glucose effects, as suggested by previous studies. The present study has focused on the role of rostral VLM CA neurons in glucoregulation and HAAF. Since the rostral VLM CA neurons project to the spinal cord and mediate the adrenal medullary response to glucoprivation, we did not measure blood corticosterone levels in this study. However, as we injected DREADD AAV into both C1r and C1m (which has neurons projecting to the hypothalamus and regulates corticosterone release), we cannot conclusively rule out a role for corticosterone in HAAF following repeated activation of CA neurons at this rostral/medial site. Detailed studies are needed to examine the effects of long-term repeated corticosterone exposure on rostral VLM CA neuron activity and adrenomedullary epinephrine and glucose release. The effect of long-term epinephrine exposure on feeding and corticosterone release also merits further investigation. Such studies will expand our knowledge about the pathogenesis and clinical treatments against HAAF.

In the present study, repeated CNO injection decreased 2DG-induced Fos expression on the non-AAV C1 side as well. This result suggests potential functional inputs between bilateral C1 CA neurons. Supporting this idea, we observed sparse mCherry-positive fibers in the contralateral C1 region following ipsilateral AAV-DREADD injection. This finding aligns with previous reports in which a few mCherry fibers were detected in the contralateral RVLM after AAV2-DIO-EF1α-ChR2-mCherry injection into the ipsilateral RVLM region in mice (39), and specifically, in which a few enhanced green fluorescent protein fibers were found in the contralateral RVLM after a lentivirus expressing enhanced green fluorescent protein under the control of DBH promotor injection into the ipsilateral RVLM region in rats (40). Indeed, chemical or electrical activation of RVLM bulbospinal neurons has been shown to modulate spontaneous activity of neurons in the contralateral RVLM (41). A study using retrograde tracers has indicated that several phenotypes of neurons directly project to the contralateral RVLM, with only a small percentage of these being CA neurons (42). Consequently, further investigation is required to determine whether this crosstalk is mediated by direct or indirect inputs between bilateral C1 CA neurons.

In summary, we found that repeated chemogenetic activation of rostral C1 CA neurons significantly reduced the CRRs to subsequent injections of either CNO or the antiglycolytic agent 2DG. The blunted response included reductions in 1) adrenal medullary epinephrine release, 2) hyperglycemia, and 3) Fos expression both in VLM CA neurons and in the adrenal medulla. Taken together our data strongly suggest that repeated stimulation of VLM CA neurons is a fundamental cause of the impairment of CRR elicitation associated with the pathogenesis of HAAF disorder.

## Data Availability

All data generated or analyzed during this study are included in this published article.

## Abbreviations

2DG: 2-deoxy-D-glucose
AAV: adeno-associated virus
CA: catecholamine
CNO: clozapine-N-oxide
CRRs: counterregulatory responses
DBH: dopamine-β hydroxylase
DREADD: designer receptor exclusively activated by designer drugs
HAAF: hypoglycemia-associated autonomic failure
IHC: immunohistochemical
NHS: normal horse serum
PBS: phosphate-buffered saline
RVLM: rostral ventrolateral medulla
Sal: saline
TH: tyrosine hydroxylase
VLM: ventrolateral medulla
